# Investigation of a time-fractional COVID-19 mathematical model with singular kernel

**DOI:** 10.1186/s13662-022-03701-z

**Published:** 2022-04-18

**Authors:** Adnan, Amir Ali, Mati ur Rahmamn, Zahir Shah, Poom Kumam

**Affiliations:** 1 Department of Mathematics, University of Malakand, Dir(L), Khyber Pakhtunkhwa Pakistan; 2 Department of Mathematics, Shanghai Jiao Tong University, 800 Dongchuan Road, Shanghai, P.R. China; 3 Department of Mathematical Sciences, University of Lakki Marwat, Lakki Marwat, 28420 Khyber Pakhtunkhwa Pakistan; 4 Fixed Point Research Laboratory, Fixed Point Theory and Applications Research Group, Center of Excellence in Theoretical and Computational Science (TaCS-CoE), Faculty of Science, King Mongkut’s University of Technology Thonburi (KMUTT), 126 Pracha Uthit Rd., Bang Mod, Thung Khru, Bangkok, 10140 Thailand; 5 Department of Medical Research, China Medical University Hospital, China Medical University, Taichung, 40402 Taiwan

**Keywords:** Mathematical model of COVID-19, Ulam–Hyers stability, Laplace–Adomian decomposition method, Homotopy perturbation method, Caputo fractional operator

## Abstract

We investigate the fractional dynamics of a coronavirus mathematical model under a Caputo derivative. The Laplace–Adomian decomposition and Homotopy perturbation techniques are applied to attain the approximate series solutions of the considered system. The existence and uniqueness solution of the system are presented by using the Banach fixed-point theorem. Ulam–Hyers-type stability is investigated for the proposed model. The obtained approximations are compared with numerical simulations of the proposed model as well as associated real data for numerous fractional-orders. The results reveal a good comparison between the numerical simulations versus approximations of the considered model. Further, one can see good agreements are obtained as compared to the classical integer order.

## Introduction

Diseases like a pandemic, from the Antonine Plague to the new pandemic COVID-19, have always been disastrous to humans [1]. The official public-service announcement on Coronavirus from the World Health Organization (WHO), comprising of 219 countries and territories all over the world, has reported a total number of 184,661,246 people affected by COVID-19 that begin in Wuhan (China). Of these, approximately 3 995 158 individuals have died, while 168,981,823 have recovered [2]. An epidemic has particularly an immediate rise in cases, mostly when it is only affecting the moderately localized areas, while a pandemic disease is an epidemic that spreads out in a whole country, continent, or globally, and it has chronic effects on all the population of the region [3, 4].

The viruses that spread COVID-19 develop initially via saliva drops, when an infected person coughs, sneezes or talks and also gets infected when a contaminated surface is touched and then that person touches some parts of the body before washing hands [5]. Most people infected with coronavirus feel light to medium breathing difficulty and recover without proper treatment, while the aged, or those people having a medical history, like a cardiac problem, chronic breathing and diabetes as well as some other symptoms, may develop chronic illness.

In early 1960, the coronavirus in humans was identified as the main cause for upper respiratory-tract infections in children and later diagnosed in the human embryonic larynx, its samples were obtained from the breathing tract of mature humans with a common cold [6].

In 2003, five new coronaviruses were diagnosed with the critical risky respiratory syndrome coronavirus, which was responsible for serious harm in humans [7]. The early mentioned *group I* coronavirus $NL61$ with NL and NH (New Haven) coronavirus has been notable worldwide. Effectively, these viruses are responsible for upper and lower respiratory-tract diseases and are expected to be a common human plague [8]. A severe acute respiratory syndrome, called SARS emerged in 2002–2003 as a coronavirus from southern China and spread around the world in a very short time. Over the past twenty years, several viral epidemic diseases such as SARS-CoV (2002–2003) and H1N1 influenza (2009) have been recorded. The Director-General of WHO declared the disease caused by the new CoV named COVID-19, latterly on February 11, 2020, which is the synonym of “coronavirus disease 2019”. Since its naming coronavirus has caused great concern all over the world in 2020. According to scientists, this class of virus spreads with the passage of time continuously, and SARS-CoV-2, known as COVID-19, is the same class of virus. Currently, researchers have identified seven different types of coronavirus, four of which come from the class of these viruses and transfer through the population frequently for a long period of time.

In controlling the flow rate and spread of infections in humans, careful examination of different types of diseases plays a vital role. However, in addition to infection transmission, the development of the correct strategy is also very useful. Mathematical modeling plays a vital role to control these infectious diseases and has also an important role in the determination of the dynamical flow and helps to build a useful technique for the treatment of harmful diseases. Several mathematical models are investigated to study different types of infectious diseases [9–13]. Here, we consider the new COVID-19 model having four subclasses namely; the susceptible compartment $\mathbf{S}_{h}(t)$, the infected compartment $\mathbf{I}_{h}(t)$, the recovered class $\mathbf{R}_{h}(t)$, and $\mathbf{W}(t)$ the reservoir compartment:

1 $$ \textstyle\begin{cases} \frac{d\mathbf{S}_{h}(t)}{dt}=\Pi ^{*}- \bigl(\beta _{1}^{*}\mathbf{I}_{h}(t)+ \beta _{2}^{*}\mathbf{W}(t)+d^{*}\bigr) \mathbf{S}_{h}(t), \\ \frac{d\mathbf{I}_{h}(t)}{dt}=\bigl(\beta _{1}^{*}\mathbf{I}_{h}(t)+ \beta _{2}^{*}\mathbf{W}(t)+d^{*}\bigr) \mathbf{S}_{h}(t)-\bigl(\sigma ^{*}+d^{*}+d_{1}^{*} \bigr) \mathbf{I}_{h}(t), \\ \frac{d\mathbf{R}_{h}(t)}{dt}=\sigma ^{*}\mathbf{I}_{h}(t)-d^{*} \mathbf{R}_{h}(t), \\ \frac{d\mathbf{W}(t)}{dt}=\alpha ^{*}\mathbf{I}_{h}(t)-\eta ^{*} \mathbf{W}(t), \end{cases} $$

where the used parameters in the above model with the whole description as $\Pi ^{*}$ is the rate of newborn individuals, $\beta _{1}^{*}$, $\beta _{2}^{*}$ are the rates of disease transmission, $d^{*}$ is the natural mortality rate, $d^{*}_{1}$ is the death rate, $\sigma ^{*}$ is the rate of recovery, $\eta ^{*}$ is the virus removing rate, while $\alpha ^{*}$ is the contributed ratio of the virus into the seafood market.

Here, first, we calculate the solution of the model (1) that is bounded. Suppose $N(t)$ represents the number of individuals at time *t*. Taking derivatives w.r.t. *t* of $N(t)$ and exploiting values from the above model, we obtain

$$ \frac{dN(t)}{dt}-dN\leq \Pi ^{*}.$$

The initial conditions $\mathbf{S}_{h}(0)\geq 0$, $\mathbf{I}_{h}(0)\geq 0$, $\mathbf{R}_{h}(0)\geq 0$, $\mathbf{W}(0)\geq 0$ and $N(0)=N_{0}$ give the solution of this equation in the form of

$$ N(t)\leq \frac{\Pi ^{*}}{d^{*}}+\biggl(N_{0}-\frac{\Pi ^{*}}{d^{*}} \biggr)e^{-d^{*}t}.$$

This is a bounded solution when *t* rises without bound.

The probable uniform phases (i.e., the disease-free (DFE), and the endemic) and the external forces through utilizing the use parameters in the model (basic reproductive number) along with comprehensive qualitative analysis have been discussed for the above model (1) as follows:

2 $$ \begin{aligned} &(\mathbf{S}_{h0},0,0,0,0)= \frac{\Pi ^{*}}{d^{*}}, \\ &R_{0}=\frac{\beta _{1}\Pi ^{*}}{d^{*}(\sigma ^{*}+d^{*}+d_{1}^{*})}+ \frac{\alpha ^{*}\beta _{2}^{*}\Pi ^{*}}{\eta ^{*} d^{*}(\sigma ^{*}+d^{*}+d_{1}^{*})}, \\ &\mathbf{S}_{h}^{*}= \frac{\eta ^{*}(\sigma ^{*}+d^{*}+d_{1}^{*})}{\eta ^{*}\beta _{1}^{*} +\alpha ^{*}\beta _{2}^{*}}, \\& \mathbf{I}_{h}^{*}= \frac{\eta ^{*} d^{*}(\sigma ^{*}+d^{*}+d_{1}^{*})(R_{0}-1)}{\sigma ^{*}\beta _{1}^{*}\eta ^{*}+\sigma ^{*}\beta _{2}^{*}\alpha ^{*}+d^{*}\beta _{1}^{*}\eta ^{*}+d^{*}\beta _{2}^{*}\alpha ^{*}+d_{1}^{*}\beta _{1}^{*}\eta ^{*}+d_{1}^{*}\beta _{2}^{*}\alpha ^{*}}, \\ &\mathbf{R}_{h}^{*}=\frac{\sigma ^{*}}{d^{*}}I_{h}^{*}, \\ & \mathbf{W}^{*}= \frac{\alpha ^{*}\eta ^{*}(\sigma ^{*}+d^{*}+d_{1}^{*})(R_{0}-1)}{\sigma ^{*}\beta _{1}^{*}\eta ^{*}+\sigma ^{*}\beta _{2}^{*}\alpha ^{*}+d^{*}\beta _{1}^{*}\eta ^{*}+d^{*}\beta _{2}^{*}\alpha ^{*}+d_{1}^{*}\beta _{1}^{*}\eta ^{*}+d_{1}^{*}\beta _{2}^{*}\alpha ^{*}}. \end{aligned} $$

Over the last few decades, it was found that fractional-order differential equations (FODEs) are to be investigated to enhance a real-world phenomenon with a comparable degree of efficiency. For more explanation, FDEs are applicable in medical and physical sciences, engineering, control systems, banking, and epidemiology [14–16]. They have been extensively studied in the modeling of real-world phenomena due to their valuable aspects as compared to IDEs [17, 18].

Several mathematical models are investigated at fractional-order, which are non-Gaussian and non-Markovian in nature [19–21]. There are many techniques for solving classical calculus to fractional-order through modeling by using various methods such as the Laplace–Adomian decomposition method (LADM) and the Homotopy perturbation method (HPM) of variation [22–27]. Further, the computational methods, such as the residual power series, Fourier transform, spectral methods together with some other methods, are extensively applied to study differential equations in both fractional and classical order [28–34].

The fractional operators are extensively applied in biological models of infectious disease, particularly in the field of continuous-time modeling. The most notable definitions in the fractional differential operators are those given by Riemann–Liouville, Caputo, Caputo–Fabrizio (CF), and Atangana–Baleanu in the sense of Caputo (ABC), where each operator has its own characteristics. It has been noted that the fractional derivatives are suitable tools in expressing sensitivity, physics, polymeric chemistry, linear viscoelasticity, and other fields of science [35, 36].

The main objective of this work is to investigate the newly constructed time-fractional model of COVID-19 under the Caputo operator. The considered model is successfully examined by using the well-known techniques of LADM and HPM for the series solution. Therefore, in light of the above discussion, we study the proposed model via the Caputo derivative with fractional-order $0< p\leq 1$

3 $$\begin{aligned}& \textstyle\begin{cases} {}^{\mathcalligra{C}}\mathcal{D}^{p} \mathbf{S}_{h}(t) =\Pi ^{*}-\bigl(\beta _{1}^{*} \mathbf{I}_{h}(t)+\beta _{2}^{*} \mathbf{W}(t)+d^{*}\bigr)\mathbf{S}_{h}(t), \\ {}^{\mathcalligra{C}}\mathcal{D}^{p}\mathbf{I}_{h}(t) =\bigl(\beta _{1}^{*} \mathbf{I}_{h}(t)+\beta _{2}^{*}\mathbf{W}(t)+d^{*}\bigr) \mathbf{S}_{h}(t)-\bigl( \sigma ^{*}+d^{*}+d_{1}^{*} \bigr)\mathbf{I}_{h}(t), \\ {}^{\mathcalligra{C}}\mathcal{D}^{p}\mathbf{R}_{h}(t) =\sigma ^{*}\mathbf{I}_{h}(t)-d^{*} \mathbf{R}_{h}(t), \\ {}^{\mathcalligra{C}}\mathcal{D}^{p}\mathbf{W}(t) =\alpha ^{*} \mathbf{I}_{h}(t)- \eta ^{*}\mathbf{W}(t), \end{cases}\displaystyle \end{aligned}$$

with initial conditions

$$ \mathbf{S}_{h}(0)\geq 0,\qquad \mathbf{I}_{h}(0)\geq 0,\qquad \mathbf{R}_{h}(0) \geq 0,\qquad \mathbf{W}(0)\geq 0.$$

The paper is arranged as follows. Section 1 is devoted to the introduction of the pandemic disease COVID-19 and fractional calculus. In Sect. 2, we discuss some fundamental results related to fractional calculus. In Sect. 3, the existence and uniqueness of the solution are derived for the considered model using the fixed-point approach. Further, the UH stability is shown in the same section. We construct the general series solutions for the proposed model by using LADM and HPM in Sect. 4. Numerical results and discussion are included in Sect. 5. Finally, we conclude the article in Sect. 6.

## Preliminaries

Some definitions are recalled from [37, 38].

### Definition 1

For a function $\ell \in L^{1}([0,\infty ), R)$, the Riemann–Liouville fractional integral of order *p* is given by:

$$\begin{aligned} I^{p}_{t}\ell (t)=\frac{1}{\Gamma (p)} \int _{0}^{\infty }\frac{\ell (\eta )}{(t-\eta )^{1}-p}\,d\eta ,\quad p>0, \end{aligned}$$

where the integral of the R.H.S. exists.

### Definition 2

The Caputo fractional-order derivative of order *p* is given as:

$$\begin{aligned} {}^{\mathcalligra{C}}\mathcal{D}^{p}_{t}\ell (t)=\frac{1}{\Gamma (n-p)} \int _{0}^{t} \frac{\ell (\eta )}{(t-\eta )^{n}-p-1}\ell ^{n}( \eta )\,d\eta ,\quad p>0, \end{aligned}$$

where the integral part on the R.H.S. exists and $n=[p]+1$. If $p\in (0,1)$, then one has

$$\begin{aligned} {}^{\mathcalligra{C}}\mathcal{D}^{p}_{t}\ell (t)=\frac{1}{\Gamma (1-p)} \int _{0}^{t} \frac{\ell (\eta )}{(t-\eta )^{p}}\acute{\ell }(\eta )\,d\eta . \end{aligned}$$

### Lemma 1

*In the case of FODEs*, *the following holds*

$$\begin{aligned} I^{p}\bigl[{}^{\mathcalligra{C}}\mathcal{D}^{p}_{t}\Psi \bigr](t)=\Psi (t)+\gamma _{0}+ \gamma _{1}+\gamma _{2}+\cdots+\gamma _{n-1}t^{n-1}. \end{aligned}$$

### Definition 3

In the Caputo sense, we define

$$\begin{aligned} \mathcalligra{L}\bigl[{}^{\mathcalligra{C}}\mathcal{D}^{p}_{t} r(t) \bigr]=s^{p} R(s)-\sum^{m-1}_{j=o}s^{p-j-1} r^{k}(0), \quad p \in (m-1, m), m \in \mathbb{Z}\mathbbm{^{+}}. \end{aligned}$$

### Definition 4

The equations, having linear *T* and nonlinear *N* terms, means that the homotopy perturbation techniques can be applicable, a homotopy may be constructed for a mapping $v(r, g):\Omega \times [0\times 1]\rightarrow R$

$$\begin{aligned} H(v, g)= (1-g ) \bigl[T(v)-T(u_{0}) \bigr]+g \bigl[T(v)-N(v)+f(r) \bigr]=0, \end{aligned}$$

where the fixed parameter $r\in \Omega $, $g\in [0,1]$.

## Existence and uniqueness of the proposed model

In this section, with the help of the fixed-point theorem, we show the existence and uniqueness of the system (3). The considered system (3) can be written as

4 $$\begin{aligned}& \begin{aligned} &{}^{\mathcalligra{C}}\mathbf{\mathcal{D}}_{t}^{p} \bigl(\mathbf{S}_{h}(t)\bigr) = \mathcal{K}_{1}(t, \mathbf{S}_{h},\mathbf{I}_{h},\mathbf{R}_{h}, \mathbf{W})=\Pi ^{*}-\bigl(\beta _{1}^{*} \mathbf{I}_{h}(t)+\beta _{2}^{*} \mathbf{W}(t)+d^{*}\bigr)\mathbf{S}_{h}(t), \\ &{}^{\mathcalligra{C}}\mathbf{\mathcal{D}}_{t}^{p}\bigl( \mathbf{I}_{h}(t)\bigr) = \mathcal{K}_{2}(t, \mathbf{S}_{h},\mathbf{I}_{h},\mathbf{R}_{h}, \mathbf{W}) \\ &\hphantom{{}^{\mathcalligra{C}}\mathbf{\mathcal{D}}_{t}^{p}\bigl( \mathbf{I}_{h}(t)\bigr) }=\bigl(\beta _{1}^{*}\mathbf{I}_{h}(t)+ \beta _{2}^{*}\mathbf{W}(t)+d^{*}\bigr) \mathbf{S}_{h}(t)-\bigl(\sigma ^{*}+d^{*}+d_{1}^{*} \bigr)\mathbf{I}_{h}(t), \\ &{}^{\mathcalligra{C}}\mathbf{\mathcal{D}}_{t}^{p}\bigl( \mathbf{R}_{h}(t)\bigr) = \mathcal{K}_{3}(t, \mathbf{S}_{h},\mathbf{I}_{h},\mathbf{R}_{h}, \mathbf{W})=\sigma ^{*}\mathbf{I}_{h}(t)-d^{*} \mathbf{R}_{h}(t), \\ &{}^{\mathcalligra{C}}\mathbf{\mathcal{D}}_{t}^{p}\bigl( \mathbf{W}(t)\bigr) = \mathcal{K}_{4}(t,\mathbf{S}_{h}, \mathbf{I}_{h},\mathbf{R}_{h}, \mathbf{W}) =\alpha ^{*}\mathbf{I}_{h}(t)-\eta ^{*}\mathbf{W}(t). \end{aligned} \end{aligned}$$

The system (3) is represented as

5 {DtpC(f(t))=X(t,f(t)),f(0)=f0≥0,

when

6 $$\left\{ \begin{array}{c} f(t)={\left(S_{h},I_{h},R_{h},W\right)}^{T},\\ f(0)={\left(S_{h}(0),I_{h}(0),R_{h}(0),W(0)\right)}^{T},\\ X(t,f(t))={\left(K_{i}(S_{h},I_{h},R_{h},W)\right)}^{T},\;i=1,2,3,4, \end{array}\right.$$

where $(\cdot)^{T}$ is the transpose of the vector. Next, the system (5) can be written as

7 $$f(t)=f_{0}+J^{p}X\left(t,f(t)\right)=f_{0}+\frac{1}{\Gamma (p)}\int_{0}^{t}{\left(t-\vartheta \right)}^{p-1}X\left(\vartheta ,f(\vartheta )\right)\;d\vartheta .$$

Let us suppose that the Banach space defined on an interval $[0,b]$ is a continuous function on $\mathcal{X}$ with $∥f∥=\sup_{t\in J}|f|$ and let $\mathbf{F}=C([0,b];\mathcal{X})$. Next, we use the following assumption:

$C_{1}$: There exists a constant $\mathcal{L}{\mathcal{X}}>0$ such that for each $f_{1},f_{2}\in C([J,R])$

$$|X\left(t,f_{1}(t)\right)-X\left(t,f_{2}(t)\right)|\leq LX|f_{1}(t)-f_{2}(t)|.$$

$C_{2}$: There exists a constant $\mathcal{K}\in ({C}[0,b],R)$ such that for all $(t,f)\in J\times R^{4}$, we have

|X(t,f)|≤K(t).

Now, to find whether the solution is unique, we use the following theorem.

### Theorem 1

*Using the assumption* $(C_{1})$, $\mathcal{X}\in {C}([\mathcal{J}, \mathbf{R])}$ *and with the maps* $\mathcal{J}\times {\mathbf{R}}^{4}$ *bounded subset to relatively compact subset to* **R**. *If* $q\mathcal{L}_{\mathcal{X}}<1$, *then the system* (3) *has a unique solution and* $\Theta =\frac{b^{p}}{\Gamma (p+1)}$.

### Proof

Let the operator $\mathbf{G}:\mathbf{Y}\rightarrow \mathbf{Y}$ expressed as

8 $$(Gf)(t)=f_{0}+\frac{1}{\Gamma (p)}\int_{0}^{t}{\left(t-\vartheta \right)}^{p-1}X\left(\vartheta ,f(\vartheta )\right)\;d\vartheta .$$

Equation (8) shows that the unique solution for (3) represents the fixed point of the operator **G**. Additionally, $\sup_{t\in \mathcal{J}}\|\mathcal{X}(t,0)\|=\mathcal{M}_{1}$ and $\vartheta \geq ∥f_{0}∥+pM_{1}$. Thus, it is enough to show that $\mathbf{G}\mathbf{P}{\vartheta }\subset \mathbf{P}{\vartheta }$ and the set is given by Pϑ={f∈Y:∥f∥≤ϑ} is convex and closed. Further, for every $f\in P_{\vartheta }$, we obtain

9 $$\begin{array}{rl} \left(Gf)(t\right) & \leq f_{0}+\frac{1}{\Gamma (p)}\int_{0}^{t}{\left(t-\vartheta \right)}^{p-1}|X\left(\vartheta ,f(\vartheta )\right)|\;d\vartheta \\ & \leq f_{0}+\frac{1}{\Gamma (p)}\int_{0}^{t}{\left(t-\vartheta \right)}^{p-1}\left[|X\left(\vartheta ,f(\vartheta )\right)-X(\vartheta ,0)|+|X(\vartheta ,0)|\right]\;d\vartheta \\ & \leq f_{0}+\frac{LX\vartheta +M_{1}}{\Gamma (p)}\int_{0}^{t}(t-\vartheta )\;d\vartheta \\ & \leq f_{0}+\frac{LX\vartheta +M_{1}}{\Gamma (p+1)}b^{p}\\ & \leq f_{0}+p(LX\vartheta +M_{1})\leq \vartheta . \end{array}$$

Next, for $f_{1},f_{2}\in Y$, we have

10 $$\begin{array}{rl} \left|(Gf_{1})(t)-(Gf_{2})(t)\right| & \leq \frac{1}{\Gamma (p)}\int_{0}^{t}{\left(t-\vartheta \right)}^{\left(p-1\right)}|X\left(\vartheta ,f_{1}(\vartheta )\right)-X\left(\vartheta ,f_{2}(\vartheta )\right)|\;d\vartheta \\ & \leq \frac{L_{X}}{\Gamma (p)}\int_{0}^{t}{\left(t-\vartheta \right)}^{\left(p-1\right)}|f_{1}(\vartheta )-f_{2}(\vartheta )|\;d\vartheta \\ & \leq L_{X}|f_{1}(t)-f_{2}(t)|, \end{array}$$

which shows that $∥(Gf_{1})-(Gf_{2})∥\leq LX∥f_{1}-f_{2}∥$. Therefore, by the Banach contraction principle, the system (3) has a unique solution on $\mathcal{J}$. □

Next, using the Schauder fixed-point theory, we find the existence of the solution for system (3).

### Lemma 2

*Consider a bounded*, *convex and closed subset of a Banach space* **Y** *is* $\mathcal{M}$. *Further*, *for the operator* $\mathbf{G}_{1}$, $\mathbf{G}_{2}$, *if the following holds*:

- $G_{1}f_{1}+G_{2}f_{2}$, *only if* $f_{1},f_{2}\in M$.
- *The operator* $\mathbf{G}_{1}$ *is a continuous and compact*.
- $\mathbf{G}_{2}$ *is a contraction mapping*.

*Then*, *there exists* $u\in \mathcal{M}$, *so that* $u=\mathbf{G}_{1}u+\mathbf{G}_{2} u$.

### Theorem 2

*Using the assumption* $C_{1}$ *and* $C_{2}$ *with* $\mathcal{X}:\mathcal{J}\times \mathbf{{R}^{4}\rightarrow {\mathbf{R}}}$, *the system* (3) *has at least one solution on* $\mathcal{J}$ *if*

$$L_{\vartheta }∥f_{1}(t_{0})-f_{2}(t_{0})∥<1.$$

### Proof

Suppose that $\sup_{t\in \mathcal{J}}|\mathcal{K}(t)|=\|\mathcal{K}\|$ and $∥f_{0}∥+p∥K∥\leq \rho$, with Bρ={f∈E:∥f∥≤ρ}. For every $\mathbf{G_{1}}$, $\mathbf{G}_{2}$ on **B***ρ* given as

$$(G_{1}f)(t)=\frac{1}{\Gamma (p)}\int_{0}^{t}{\left(t-\vartheta \right)}^{p-1}X\left(\vartheta ,f(\vartheta )\right)\;d\vartheta ,\;t\in J,$$

and $\left(G_{2}f)(t)=f(t_{0}\right)$, $t\in \mathcal{J}$. Hence, for all $\mathbf{G}_{1},\mathbf{G}_{2}\in \mathbf{B}{\rho }$, we obtain

11 $$\begin{array}{rl} ∥(G_{1}f_{1})(t)+(G_{2}f_{2})(t)∥ & \leq ∥f_{0}∥+\frac{1}{\Gamma (p)}\int_{0}^{t}{\left(t-\vartheta \right)}^{p-1}∥X\left(\vartheta ,G_{1}(\vartheta )\right)∥\;d\vartheta \\ & \leq ∥f_{0}∥+p∥K∥\\ & \leq \rho <\infty . \end{array}$$

Hence, $G_{1}f_{1}+G_{2}f_{2}\in B\rho$.

Next, we prove the contraction of $\mathbf{G}_{2}$.

Given that any $t\in \mathcal{J}$ and $\mathbf{G}_{1},\mathbf{G}_{2}\in \mathbf{B}{\rho }$, it gives

12 $$∥(G_{1}f_{1})(t)-(G_{2}f_{2})(t)∥\leq ∥G_{1}(t_{0})-G_{2}(t_{0})∥.$$

Having a continuous function $\mathcal{X}$, thus $\mathbf{G}_{1}$ is continuous. Moreover, for all $t\in \mathcal{J}$ and $f_{1}\in B\rho$,

$$∥G_{1}f∥\leq ∥K∥<\infty .$$

Hence, $\mathbf{G}_{1}$ is bounded uniformly. Finally, we show $\mathbf{G}_{1}$ is compact. Let us suppose that $\sup(t,f)\in (J\times B_{\rho })|X(\vartheta ,f(\vartheta ))|=X^{*}$, which gives

13 $$\begin{array}{c} \left|(G_{1}f)(t_{2})-(G_{1}f)(t_{1})\right|\\ \;=\frac{1}{\Gamma (p)}|\int_{0}^{t}\left[{\left(t_{2}-\vartheta \right)}^{p-1}-{\left(t_{1}-\vartheta \right)}^{p-1}\right]X\left(\vartheta ,f(\vartheta )\right)\;d\vartheta +\int_{1}^{2}{\left(t_{2}-\vartheta \right)}^{p-1}X\left(\vartheta ,f(\vartheta )\right)|\\ \;\leq \frac{X^{*}}{\Gamma (p)}\left[2{\left(t_{2}-t_{1}\right)}^{p}+\left(t_{2}^{p}-t_{1}^{p}\right)\right]\to 0\;\text{as }t_{2}\to t_{1}. \end{array}$$

In view of the well-known “Arzela–Ascoli theorem”, the operator $\mathbf{G}_{1}$ is relatively compact on **B***ρ* and thus $\mathbf{G}_{1}$ is completely continuous. All the claims of Lemma 2 are satisfied, therefore, we deduce that the proposed system (3) has at least one solution. □

### Ulam–Hyers stability

In this section, we discuss the UH stability and generalized UH stability [27, 39] for the proposed model. Let us assume *ϵ* with the following inequality:

14 $$|{}^{C}D^{p}\tilde{f}(t)-X\left(t,\tilde{f}(t)\right)|\leq \epsilon ,\;t\in J,\epsilon =\max{\left(\epsilon_{i}\right)}^{T},i=1,2,3,4.$$

#### Definition 5

System (3) is UH stable if there exist $\mathbf{U}_{\mathcal{X}}>0$ for all $\epsilon >0$, solution of $\tilde{f}\in Y$ holds for Eq. (14), there is a unique solution f∈Y for Eq. (5) such that

$$|\tilde{f}(t)-f(t)|\leq U_{X}\epsilon ,\;t\in J,\;UX=\max{\left(UX_{j}\right)}^{T}.$$

#### Definition 6

System (3) is generalized UH stable if there exists a continuous function $\Phi : R^{+}\rightarrow R^{+}$ and $\Phi (0)=0$, so that for all solution $\tilde{f}\in Y$ of Eq. (14), then there is a unique solution f∈Y for Eq. (5) with the following:

$$|\tilde{f}(t)-f(t)|\leq \Phi_{X}\epsilon ,\;t\in J,\;\Phi_{X}=\max{\left(\Phi_{X_{j}}\right)}^{T}.$$

#### Remark 1

A function $\tilde{f}\in Y$ satisfies Eq. (14) if and only if there exists a function $\Phi \in \mathbf{Y}$ with the following properties:

(I) $|\Phi (t)|\leq \epsilon $, $\Phi =\max (\Phi _{j})$, $t\in \mathcal{J}$.
(II) DpCf˜(t)=X(t,f˜(t))+Φ(t), $t\in \mathcal{J}$.

#### Lemma 3

*If* $\tilde{f}\in Y$ *holds for Eq*. (14), *then* $\tilde{f}$ *also holds for the following*

15 $$|\tilde{f}(t)-{\tilde{f}}_{0}(t)-\frac{1}{\Gamma (p)}\int_{0}^{t}{\left(t-\vartheta \right)}^{p-1}X\left(\vartheta ,\tilde{f}(\vartheta )\right)\;d\vartheta |\leq \epsilon .$$

#### Proof

Using (1), we have

DpCf˜(t)=X(t,f˜(t))+Φ(t),

along with Lemma (3), we obtain

16 $$\tilde{f}(t)={\tilde{f}}_{0}(t)+\frac{1}{\Gamma (p)}\int_{0}^{t}{\left(t-\vartheta \right)}^{p-1}X\left(\vartheta ,\tilde{f}(\vartheta )\right)\;d\vartheta +\frac{1}{\Gamma (p)}\int_{0}^{t}{\left(t-\vartheta \right)}^{p-1}\Phi (\vartheta )\;d\vartheta .$$

Next, using (1) gives

17 $$\begin{array}{c} \left|\tilde{f}(t)-{\tilde{f}}_{0}(t)-\frac{1}{\Gamma (p)}\int_{0}^{t}{\left(t-\vartheta \right)}^{p-1}X\left(\vartheta ,\tilde{f}(\vartheta )\right)\;d\vartheta \right|\\ \;\leq \frac{1}{\Gamma (p)}\int_{0}^{t}{\left(t-\vartheta \right)}^{p-1}|\Phi (\vartheta )|\;d\vartheta \leq \epsilon . \end{array}$$

Thus, the proof is completed. □

#### Theorem 3

*For all* f∈Y *and* $\mathcal{X}:\mathcal{J}\times R^{4}\rightarrow R$ *with the assumption* $({C}_{1})$ *holds and* $1-p\mathcal{L}{\mathcal{X}}>0$. *Equation* (5) *is equal to Eq*. (3) *and is UH stable and consequently*, *generalized UH stable*.

#### Proof

Suppose that $f,\tilde{f}\in Y$ is a unique solution of Eq. (5), therefore for all $\epsilon >0$, $t\in \mathcal{J}$ along with Lemma 3, we have

$$\begin{array}{rcl} \left|\tilde{f}(t)-f(t)\right| & = & \max_{t\in J}|\tilde{f}(t)-f_{0}-\frac{1}{\Gamma (p)}\int_{0}^{t}{\left(t-\vartheta \right)}^{p-1}X\left(\vartheta ,f(\vartheta )\right)\;d\vartheta |\\ & \leq & \max_{t\in J}|\tilde{f}(t)-{\tilde{f}}_{0}-\frac{1}{\Gamma (p)}\int_{0}^{t}{\left(t-\vartheta \right)}^{p-1}X\left(\vartheta ,f(\vartheta )\right)\;d\vartheta |\\ & & +\max_{t\in J}\frac{1}{\Gamma (p)}\int_{0}^{t}{\left(t-\vartheta \right)}^{p-1}|X\left(\vartheta ,\tilde{f}(\vartheta )\right)-X\left(\vartheta ,f(\vartheta )\right)|\;d\vartheta \\ & \leq & \left|f(t)-{\tilde{f}}_{0}-\frac{1}{\Gamma (p)}\int_{0}^{t}{\left(t-\vartheta \right)}^{p-1}X\left(\vartheta ,\tilde{f}(\vartheta )\right)\;d\vartheta \right|\\ & & +\frac{L_{X}}{\Gamma (p)}\int_{0}^{t}{\left(t-\vartheta \right)}^{p-1}|\tilde{f}(\vartheta )-f(\vartheta )|\;d\vartheta \\ & \leq & p\epsilon +pL_{X}|\tilde{f}(t)-f(t)|, \end{array}$$

from which we have

18 $$∥\tilde{f}-f∥\leq U_{X}\epsilon .$$

From Eq. (18), we may write

19 $$ \mathbf{U} {\mathcal{X}}=\frac{p}{1-p\mathcal{L}{\mathcal{X}}}. $$

Hence, equating $\Phi _{\mathcal{X}}(\epsilon )=\mathbf{U}{\mathcal{X}}\epsilon $, so that $\Phi {\mathcal{X}}(0)=0$, we conclude that the solution of Eq. (3) is stable for both UH and generalized UH. □

## Analytical approach to the proposed model

In this section, we study the approximate series solutions of the proposed model using LADM and HPM methods.

### General solution of the proposed system by using LADM

Here, we study the general approach for the proposed model (3) subject to initial conditions. Taking Laplace transforms of both sides of the proposed model (3), we obtain

20 $$\begin{aligned} \mathcalligra{L} \bigl[ {}^{\mathcalligra{C}}\mathcal{D}_{t}^{p} \mathbf{S}_{h}(t) \bigr] =&\mathcalligra{L} \bigl[\Pi ^{*}-\bigl( \beta _{1}^{*}\mathbf{I}_{h}(t) + \beta _{2}^{*}\mathbf{W}(t)+d^{*}\bigr) \mathbf{S}_{h}(t) \bigr], \\ \mathcalligra{L} \bigl[ {}^{\mathcalligra{C}}\mathcal{D}_{t}^{p} \mathbf{I}_{h}(t) \bigr] =&\mathcalligra{L} \bigl[\bigl(\beta _{1}^{*}\mathbf{I}_{h}(t)+\beta _{2}^{*} \mathbf{W}(t)+d^{*}\bigr) \mathbf{S}_{h}(t)- \bigl(\sigma ^{*}+d^{*}+d_{1}^{*} \bigr) \mathbf{I}_{h}(t) \bigr], \\ \mathcalligra{L} \bigl[ {}^{\mathcalligra{C}}\mathcal{D}_{t}^{p} \mathbf{R}_{h}(t) \bigr] =&\mathcalligra{L} \bigl[\sigma ^{*} \mathbf{I}_{h}(t)-d^{*}\mathbf{R}_{h}(t) \bigr], \\ \mathcalligra{L} \bigl[ {}^{\mathcalligra{C}}\mathcal{D}_{t}^{p} \mathbf{W}(t) \bigr] =& \mathcalligra{L} \bigl[\alpha ^{*} \mathbf{I}_{h}(t)-\eta ^{*}\mathbf{W}(t) \bigr]. \end{aligned}$$

Applying initial conditions (20), we obtain

21 $$\begin{aligned}& \mathcalligra{L} \bigl[\mathbf{S}_{h}(t) \bigr] = \frac{M_{1}}{s}+ \frac{1}{s^{p}}\mathcalligra{L} \bigl[\Pi ^{*}-\bigl( \beta _{1}^{*}\mathbf{I}_{h}(t) +\beta _{2}^{*}\mathbf{W}(t)+d^{*}\bigr)\mathbf{S}(t) \bigr], \\& \mathcalligra{L} \bigl[\mathbf{I}_{h}(t) \bigr] = \frac{M_{2}}{s}+ \frac{1}{s^{p}}\mathcalligra{L} \bigl[\bigl(\beta _{1}^{*} \mathbf{I}_{h}(t) + \beta _{2}^{*} \mathbf{W}(t)+d^{*}\bigr)\mathbf{S}(t)-\bigl(\sigma ^{*}+d^{*}+d_{1}^{*} \bigr) \mathbf{I}_{h}(t) \bigr], \\& \mathcalligra{L} \bigl[\mathbf{R}_{h}(t) \bigr] = \frac{M_{3}}{s}+ \frac{1}{s^{p}}\mathcalligra{L} \bigl[\sigma ^{*}\mathbf{I}_{h}(t)-d^{*} \mathbf{R}_{h}(t) \bigr], \\& \mathcalligra{L} \bigl[\mathbf{W}(t) \bigr] = \frac{M_{4}}{s}+\frac{1}{s^{p}} \mathcalligra{L} \bigl[\alpha ^{*}\mathbf{I}_{h}(t)-\eta ^{*}\mathbf{W}(t) \bigr]. \end{aligned}$$

Suppose the series solutions of **S**, **I**, **R** and **W** up to infinite terms are

22 $$\begin{aligned}& \begin{aligned} &{\mathbf{S}}(t)=\sum^{\infty }_{n=0} \mathbf{S}_{n}(t), \qquad \mathbf{I}(t)=\sum ^{\infty }_{n=0}\mathbf{I}_{n}(t), \\ &\mathbf{R}(t)=\sum^{\infty }_{n=0} \mathbf{R}_{n}(t), \qquad \mathbf{W}(t)=\sum ^{\infty }_{n=0}\mathbf{W}_{n}(t), \end{aligned} \end{aligned}$$

where $\mathbf{S}_{h}(t)\mathbf{I}_{h}(t)=\sum^{\infty }_{n=0} \mathbf{P}_{n}(t)$, and $\mathbf{S}_{h}(t)\mathbf{W}(t)=\sum^{\infty }_{n=0}\mathbf{Q}_{n}(t)$ can be decomposed in the form of the Adomian polynomial

23 $$\begin{aligned}& \begin{aligned} &\mathbf{P}_{n}(t)= \frac{1}{n!} \frac{d^{n}}{d\lambda ^{n}} \Biggl[\sum_{k=0}^{n} \lambda ^{k} \mathbf{S}_{k}(t)\sum _{k=0}^{n} \lambda ^{k} \mathbf{I}_{k}(t) \Biggr]_{\lambda =0}, \\ &\mathbf{Q}_{n}(t)=\frac{1}{n!}\frac{d^{n}}{d\lambda ^{n}} \Biggl[\sum _{k=0}^{n}\lambda ^{k}\mathbf{S}_{k}(t)\sum_{k=0}^{n} \lambda ^{k}\mathbf{W}_{k}(t) \Biggr]_{\lambda =0}. \end{aligned} \end{aligned}$$

Putting Eqs. (22) and (23) into Eq. (21) and comparing like terms on each sides, we obtain:

24 $$\begin{aligned}& \mathcalligra{L}\bigl[\mathbf{S}_{0}(t)\bigr]=\frac{\mathrm{M}_{1}}{s},\qquad \mathcalligra{L}\bigl[{ \mathbf{I}_{0}(t)}\bigr]= \frac{\mathrm{M}_{2}}{s}, \\& \mathcalligra{L}\bigl[ {\mathbf{R}_{0}(t)}\bigr]=\frac{\mathrm{M}_{3}}{s},\qquad \mathcalligra{L}\bigl[ {\mathbf{W}_{0}(t)}\bigr]=\frac{\mathrm{M}_{4}}{s}, \\& \mathcalligra{L}\bigl[\mathbf{S}_{1}(t)\bigr]=\frac{1}{s^{p}} \mathcalligra{L} \bigl[\Pi ^{*}-\bigl(\beta _{1}^{*} \mathbf{P}_{0}+\beta _{2}^{*} \mathbf{Q}_{0}\bigr)+d^{*}\mathbf{S}_{0} \bigr], \\& \mathcalligra{L}\bigl[\mathbf{I}_{1}(t)\bigr]=\frac{1}{s^{p}} \mathcalligra{L} \bigl[\bigl(\beta _{1}^{*}\mathbf{P}_{0}+ \beta _{2}^{*}\mathbf{Q}_{0}+d^{*} \mathbf{S}_{0}\bigr)-\bigl(\sigma ^{*}+d^{*}+d_{1}^{*} \bigr)\mathbf{I}_{0} \bigr], \\& \mathcalligra{L}\bigl[{\mathbf{R}_{1}(t)}\bigr]=\frac{1}{s^{p}} \mathcalligra{L} \bigl[\sigma ^{*}\mathbf{I}_{0}-d^{*} \mathbf{R}_{0} \bigr], \\& \mathcalligra{L}\bigl[{\mathbf{W}_{1}(t)}\bigr]=\frac{1}{s^{p}} \mathcalligra{L} \bigl[\alpha ^{*}\mathbf{I}_{0}-\eta ^{*}\mathbf{W}_{0} \bigr], \\& \mathcalligra{L}\bigl[{\mathbf{S}_{2}(t)}\bigr]= \frac{1}{s^{p}}\mathcalligra{L} \bigl[\Pi ^{*}-\bigl(\beta _{1}^{*}\mathbf{P}_{1}+\beta _{2}^{*} \mathbf{Q}_{1}\bigr)+d^{*}\mathbf{S}_{1} \bigr], \\& \mathcalligra{L}\bigl[{\mathbf{I}_{2}(t)}\bigr]= \frac{1}{s^{p}}\mathcalligra{L} \bigl[\bigl(\beta _{1}^{*} \mathbf{P}_{1}+\beta _{2}^{*}\mathbf{Q}_{1}+d^{*} \mathbf{S}_{1}\bigr)-\bigl(\sigma ^{*}+d^{*}+d_{1}^{*} \bigr)\mathbf{I}_{1} \bigr], \\& \mathcalligra{L}\bigl[{\mathbf{R}_{2}(t)}\bigr]= \frac{1}{s^{p}}\mathcalligra{L} \bigl[\sigma ^{*}\mathbf{I}_{1}-d^{*} \mathbf{R}_{1} \bigr], \\& \mathcalligra{L}\bigl[{\mathbf{W}_{2}(t)}\bigr]= \frac{1}{s^{p}}\mathcalligra{L} \bigl[\alpha ^{*}\mathbf{I}_{1}- \eta ^{*}\mathbf{W}_{1} \bigr], \\& \vdots \\& \mathcalligra{L}\bigl[{\mathbf{S}_{n+1}(t)}\bigr]= \frac{1}{s^{p}} \mathcalligra{L} \bigl[\Pi ^{*}-\bigl(\beta _{1}^{*}\mathbf{P}_{n}+\beta _{2}^{*} \mathbf{Q}_{n}\bigr)+d^{*}\mathbf{S}_{n} \bigr], \\& \mathcalligra{L}\bigl[{\mathbf{I}_{n+1}(t)}\bigr]= \frac{1}{s^{p}} \mathcalligra{L} \bigl[\bigl(\beta _{1}^{*} \mathbf{P}_{n}+\beta _{2}^{*}\mathbf{Q}_{n}+d^{*} \mathbf{S}_{n}\bigr)-\bigl(\sigma ^{*}+d^{*}+d_{1}^{*} \bigr)\mathbf{I}_{n} \bigr], \\& \mathcalligra{L}\bigl[{\mathbf{R}_{n+1}(t)}\bigr]= \frac{1}{s^{p}} \mathcalligra{L} \bigl[\sigma ^{*}\mathbf{I}_{n}-d^{*} \mathbf{R}_{n} \bigr], \\& \mathcalligra{L}\bigl[{\mathbf{W}_{n+1}(t)}\bigr]= \frac{1}{s^{p}} \mathcalligra{L} \bigl[\alpha ^{*}\mathbf{I}_{n}- \eta ^{*}\mathbf{W}_{n} \bigr]. \end{aligned}$$

Now, using the inverse Laplace transform of Eq. (24), we have

25 $$\begin{aligned}& \bigl[{\mathbf{S}_{0}(t)} \bigr]=\mathcalligra{L}^{-1} \biggl[ \frac{\mathrm{M}_{1}}{s} \biggr],\qquad \mathcalligra{L}^{-1}\bigl[ {\mathbf{I}_{0}(t)}\bigr]= \biggl[\frac{\mathrm{M}_{2}}{s} \biggr], \\ & \bigl[{\mathbf{R}_{0}(t)} \bigr]=\mathcalligra{L}^{-1} \biggl[ \frac{\mathrm{M}_{3}}{s} \biggr],\qquad \bigl[ {\mathbf{W}_{0}(t)}\bigr]= \mathcalligra{L}^{-1} \biggl[ \frac{\mathrm{M}_{4}}{s} \biggr], \\ & \bigl[\mathbf{S}_{1}(t)\bigr]=\mathcalligra{L}^{-1} \biggl[\frac{1}{s^{p}} \mathcalligra{L} \bigl(\Pi ^{*}-\bigl(\beta _{1}^{*}\mathbf{P}_{0}+\beta _{2}^{*} \mathbf{Q}_{0}\bigr)+d^{*}\mathbf{S}_{0} \bigr) \biggr], \\ & \bigl[\mathbf{I}_{1}(t)\bigr]=\mathcalligra{L}^{-1} \biggl[\frac{1}{s^{p}} \mathcalligra{L} \bigl(\beta _{1}^{*} \mathbf{P}_{0}+\beta _{2}^{*} \mathbf{Q}_{0}+d^{*}\mathbf{S}_{0}-\bigl(\sigma ^{*}+d^{*}+d_{1}^{*}\bigr) \mathbf{I}_{0} \bigr) \biggr], \\ & \bigl[{\mathbf{R}_{1}(t)}\bigr]=\mathcalligra{L}^{-1} \biggl[\frac{1}{s^{p}} \mathcalligra{L} \bigl(\sigma ^{*} \mathbf{I}_{0}-d^{*}\mathbf{R}_{0} \bigr) \biggr], \\ & \bigl[{\mathbf{W}_{1}(t)}\bigr]=\mathcalligra{L}^{-1} \biggl[\frac{1}{s^{p}} \mathcalligra{L} \bigl(\alpha ^{*} \mathbf{I}_{0}-\eta ^{*}\mathbf{W}_{0} \bigr) \biggr], \\ & \bigl[{\mathbf{S}_{2}(t)}\bigr]=\mathcalligra{L}^{-1} \biggl[\frac{1}{s^{p}} \mathcalligra{L} \bigl(\Pi ^{*}-\bigl(\beta _{1}^{*}\mathbf{P}_{1}+\beta _{2}^{*} \mathbf{Q}_{1}\bigr)+d^{*}\mathbf{S}_{1} \bigr) \biggr], \\ & \bigl[{\mathbf{I}_{2}(t)}\bigr]=\mathcalligra{L}^{-1} \biggl[\frac{1}{s^{p}} \mathcalligra{L} \bigl(\beta _{1}^{*} \mathbf{P}_{1}+\beta _{2}^{*} \mathbf{Q}_{1}+d^{*} \mathbf{S}_{1} -\bigl(\sigma ^{*}+d^{*}+d_{1}^{*} \bigr) \mathbf{I}_{1} \bigr) \biggr], \\ & \bigl[{\mathbf{R}_{2}(t)}\bigr]=\mathcalligra{L}^{-1} \biggl[\frac{1}{s^{p}} \mathcalligra{L} \bigl(\sigma ^{*} \mathbf{I}_{1}-d^{*}\mathbf{R}_{1} \bigr) \biggr], \\& \bigl[{\mathbf{W}_{2}(t)}\bigr]=\mathcalligra{L}^{-1} \biggl[\frac{1}{s^{p}} \mathcalligra{L} \bigl(\alpha ^{*} \mathbf{I}_{1}-\eta ^{*}\mathbf{W}_{1} \bigr) \biggr], \\& \vdots \\& \bigl[{\mathbf{S}_{n+1}(t)}\bigr]=\mathcalligra{L}^{-1} \biggl[ \frac{1}{s^{p}}\mathcalligra{L} \bigl(\Pi ^{*}-\bigl(\beta _{1}^{*}\mathbf{P}_{n}+ \beta _{2}^{*} \mathbf{Q}_{n}+d^{*}\mathbf{S}_{n}\bigr) \bigr) \biggr], \\& \bigl[{\mathbf{I}_{n+1}(t)}\bigr]=\mathcalligra{L}^{-1} \biggl[ \frac{1}{s^{p}}\mathcalligra{L} \bigl(\beta _{1}^{*} \mathbf{P}_{n}+\beta _{2}^{*} \mathbf{Q}_{n}+d^{*} \mathbf{S}_{n}\bigr) -\bigl(\sigma ^{*}+d^{*}+d_{1}^{*} \bigr) \mathbf{I}_{n} ) \biggr], \\& \bigl[{\mathbf{R}_{n+1}(t)}\bigr]=\mathcalligra{L}^{-1} \biggl[ \frac{1}{s^{p}}\mathcalligra{L} \bigl(\sigma ^{*} \mathbf{I}_{n}-d^{*} \mathbf{R}_{n} \bigr) \biggr], \\& \bigl[{\mathbf{W}_{n+1}(t)}\bigr]=\mathcalligra{L}^{-1} \biggl[ \frac{1}{s^{p}}\mathcalligra{L} \bigl(\alpha ^{*} \mathbf{I}_{n}-\eta ^{*} \mathbf{W}_{n} \bigr) \biggr], \end{aligned}$$

and after simplification of Eq. (25), we obtain

26 $$\begin{aligned}& {\mathbf{S}_{0}(t)} = \mathrm{M}_{1},\qquad { \mathbf{I}_{0}(t)}=\mathrm{M}_{2},\qquad { \mathbf{R}_{0}(t)}=\mathrm{M}_{3},\qquad { \mathbf{W}_{0}(t)}=\mathrm{M}_{4}, \end{aligned}$$

27 $$\begin{aligned}& \mathbf{S}_{1}(t) = { \bigl[\Pi ^{*}-\beta _{1}^{*}M_{1}M_{2}- \beta _{2}^{*}M_{1}M_{4}-d^{*}M_{1} \bigr]\frac{t^{p}}{\Gamma (p+1)}}, \\& \mathbf{I}_{1}(t) = { \bigl[\bigl(\beta _{1}^{*}M_{1}M_{2}+\beta _{2}^{*}M_{1}M_{4}+d^{*}M_{1} \bigr) -\bigl(\sigma ^{*}+d^{*}+d^{*}_{1} \bigr)M_{2} \bigr]\frac{t^{p}}{\Gamma (p+1)}}, \\& {\mathbf{R}_{1}(t)} = { \bigl[\sigma ^{*}M_{2}-d^{*}M_{3} \bigr] \frac{t^{p}}{\Gamma (p+1)}}, \\& {\mathbf{W}_{1}(t)} = { \bigl[\alpha ^{*}M_{2}-\eta ^{*}M_{4} \bigr] \frac{t^{p}}{\Gamma (p+1)}}, \\& {\mathbf{S}_{2}(t)} = \biggl[\Pi ^{*} \frac{t^{p}}{\Gamma (p+1)} \biggr] \\& \hphantom{{\mathbf{S}_{2}(t)} =}{}- \bigl[\beta ^{*}_{1}(k_{11}M_{1}+g_{11}M_{2}) +\beta ^{*}_{2}(v_{11}M_{1}+g_{11}M_{4})+d^{*}g_{11} \bigr]\frac{t^{2p}}{\Gamma (2p+1)}, \\& {\mathbf{I}_{2}(t)} = \bigl[\beta ^{*}_{1}(k_{11}M_{1}+g_{11}M_{2})+ \beta ^{*}_{2}(v_{11}M_{1} +g_{11}M_{4})+d^{*}g_{11} \\& \hphantom{{\mathbf{I}_{2}(t)} =}{}-\bigl(\sigma ^{*}+d^{*}+d^{*}_{1}\bigr)M_{2} \bigr]\frac{t^{2p}}{\Gamma (2p+1)}, \\& {\mathbf{R}_{2}(t)} = { \bigl[\sigma ^{*}k_{11}-d^{*}u_{11} \bigr] \frac{t^{2p}}{\Gamma (2p+1)}}, \\& {\mathbf{W}_{2}(t)} = { \bigl[\alpha ^{*}k_{11}-\eta ^{*}v_{11} \bigr] \frac{t^{2p}}{\Gamma (2p+1)}}, \end{aligned}$$

28 $$\begin{aligned}& {\mathbf{S}_{3}(t)} = \mathrm{\Pi }^{*} \frac{t^{p}}{\Gamma (p+1)}- \bigl[\beta _{1}M_{1}g_{11}k_{11}+ \beta _{1}M_{1} \Pi ^{*} +\beta _{2}v_{11}g_{11}+ \beta _{2}M_{4}+d^{*}\Pi ^{*} \bigr] \frac{t^{2p}}{\Gamma (2p+1)} \\& \hphantom{{\mathbf{S}_{3}(t)} =}{} - \bigl[\beta _{1}M_{1}k_{22}-\beta _{2}g_{22}+\beta _{2}M_{1}v_{22}- \beta _{2}M_{4}g_{22}-d^{*}g_{22} \bigr]\frac{t^{3p}}{\Gamma (3p+1)}, \\& {\mathbf{I}_{3}(t)} = { \bigl[\beta _{1}M_{1} g_{11} k_{11}}- \beta _{1}M_{1}\Pi ^{*}-\beta _{2}v_{11}g_{11}-\beta _{2}M_{4} -d^{*} \Pi ^{*} \bigr] \frac{t^{2p}}{\Gamma (2p+1)}, \\& \hphantom{{\mathbf{I}_{3}(t)} =}{} + \bigl[\beta _{1}M_{1}k_{22}+\beta _{2}g_{22}-\beta _{2}M_{1}v_{22}- \beta _{2}M_{4}g_{22}+d^{*}g_{22}- \bigl(\sigma ^{*}+d^{*}+d^{*}_{1} \bigr) \bigr]{\frac{t^{3p}}{\Gamma (3p+1)}}, \\& {\mathbf{R}_{3}(t)} = { \bigl(\sigma ^{*}k_{11}-d^{*}u_{11} \bigr)} { \frac{t^{3p}}{\Gamma (3p+1)}}, \\& \mathbf{W}_{3}(t) = { (\alpha k_{11}-\eta v_{11} )} { \frac{t^{3p}}{\Gamma (3p+1)}}. \end{aligned}$$

Similarly, we can calculate the remaining terms for the series solution of the proposed model (3).

### General solution of the proposed system by using HPM

Using the Homotopy perturbation method [40, 41] on the proposed model (3), we can write

29 $$\begin{aligned}& \textstyle\begin{cases} (1-q) \bigl[{{}^{\mathcalligra{C}}} \mathcal{D}_{t}^{p}\bigl( {\mathbf{S}_{h}(t)} \bigr)-{{}^{\mathcalligra{C}}}\mathcal{D}_{t}^{p}\bigl( \mathbf{S}_{0}(t)\bigr) \bigr] \\ \quad {}+q \bigl[{{}^{\mathcalligra{C}}} \mathcal{D}_{t}^{p}\bigl( {\mathbf{S}_{h}(t)} \bigr) -\Pi ^{*}+ \bigl(\beta _{1}^{*} \mathbf{I}_{h}(t)+ \beta _{2}^{*} \mathbf{W}(t)+d^{*} \bigr)\mathbf{S}_{h}(t) \bigr]=0, \\ (1-q)\bigl[{{}^{\mathcalligra{C}}}\mathcal{D}_{t}^{p}\bigl( {\mathbf{I}_{h}(t)}\bigr)-{}^{\mathcalligra{C}}\mathcal{D}_{t}^{p} \bigl( {\mathbf{I}_{0}(t)}\bigr)\bigr] \\ \quad {}+q \bigl[{{}^{\mathcalligra{C}}} \mathcal{D}^{p}_{t}\bigl( \mathbf{I}_{h}(t)\bigr)- \bigl(\beta _{1}^{*}\mathbf{I}_{h}(t)+\beta _{2}^{*} \mathbf{W}(t)+d^{*} \bigr) \mathbf{S}_{h}(t) \\ \quad {}+ \bigl(\sigma ^{*}+d^{*}+d_{1}^{*} \bigr)\mathbf{I}_{h}(t) \bigr]=0, \\ (1-q) \bigl[{{}^{\mathcalligra{C}}}\mathcal{D}_{t}^{p}\bigl( {\mathbf{R}_{h}(t)}\bigr)-{}^{\mathcalligra{C}}\mathcal{D}_{t}^{p} \bigl( {\mathbf{R}_{0}(t)}\bigr) \bigr] \\ \quad {}+q \bigl[{{}^{\mathcalligra{C}}} \mathcal{D}_{t}^{p}\bigl( {\mathbf{R}_{h}(t)} \bigr)+\sigma ^{*}\mathbf{I}_{h}(t)+d^{*} \mathbf{R}_{h}(t) \bigr]=0, \\ (1-q) \bigl[{{}^{\mathcalligra{C}}}\mathcal{D}_{t}^{p}\bigl( \mathbf{W}(t)\bigr)-{}^{\mathcalligra{C}}\mathcal{D}_{t}^{p} \bigl( {\mathbf{W}_{0}(t)}\bigr) \bigr] \\ \quad {}+q \bigl[{{}^{\mathcalligra{C}}} \mathcal{D}_{t}^{p}\bigl( \mathbf{W}(t)\bigr)-\alpha ^{*}\mathbf{I}_{h}(t)+\eta ^{*} \mathbf{W}(t) \bigr]=0. \end{cases}\displaystyle \end{aligned}$$

Putting $q=0$ into Eq. (29), we obtain the system of differential equations in the form

30 $$\begin{aligned} \textstyle\begin{cases} {} ^{\mathcalligra{C}}\mathcal{D}_{t}^{p} \bigl({\mathbf{S}(t)}\bigr)-{{}^{\mathcalligra{C}}}\mathcal{D}_{t}^{p} \bigl({\mathbf{S}_{0}(t)}\bigr)=0, \\ {} ^{\mathcalligra{C}}\mathcal{D}_{t}^{p}\bigl(\mathbf{I}(t)\bigr)-{{}^{\mathcalligra{C}}}\mathcal{D}_{t}^{p}\bigl( {\mathbf{I}_{0}(t)}\bigr)=0, \\ {} ^{\mathcalligra{C}}\mathcal{D}_{t}^{p}\bigl(\mathbf{R}(t)\bigr)-{{}^{\mathcalligra{C}}}\mathcal{D}_{t}^{p}\bigl( {\mathbf{R}_{0}(t)}\bigr)=0, \\ {} ^{\mathcalligra{C}}\mathcal{D}_{t}^{p}\bigl(\mathbf{W}(t)\bigr)-{{}^{\mathcalligra{C}}}\mathcal{D}_{t}^{p}\bigl( {\mathbf{W}_{0}(t)}\bigr)=0. \end{cases}\displaystyle \end{aligned}$$

For simplicity, we do not include the solution of Eq. (30). Now, inserting $q=1$ into Eq. (29), we obtain the model similar to Eq. (3). The infinite-series solution can be written as

31 $$\begin{aligned}& \begin{aligned} &{\mathbf{S}(t)}=\sum_{n=0}^{\infty }q^{n} {\mathbf{S}_{n}(t)},\qquad \mathbf{I}(t)=\sum _{n=0}^{ \infty }q^{n}{ \mathbf{I}_{n}(t)}, \\ & \mathbf{R}(t)=\sum _{n=0}^{ \infty }q^{n}{ \mathbf{R}_{n}(t)}, \qquad \mathbf{W}(t)=\sum _{n=0}^{ \infty }q^{n}{ \mathbf{W}_{n}(t)}. \end{aligned} \end{aligned}$$

Comparing like terms of *q* in Eq. (31) and Eq. (29), gives

32 $$\begin{aligned} q^{0}: \quad {\mathbf{S}_{0}(t)}= \mathrm{M}_{1},\qquad {\mathbf{I}_{0}(t)}= \mathrm{M}_{2},\qquad {\mathbf{R}_{0}(t)}= \mathrm{M}_{3},\qquad {\mathbf{W}_{0}(t)}= \mathrm{M}_{4}, \end{aligned}$$

as well as

$$\begin{aligned}& q^{1}:\textstyle\begin{cases} \mathbf{S}_{1}(t) = { [\Pi ^{*}-\beta _{1}^{*} M_{1}M_{2}-\beta _{2}^{*} M_{1}M_{4}-d^{*}M_{1} ]\frac{t^{p}}{\Gamma (p+1)}}, \\ \mathbf{I}_{1}(t) = { [(\beta _{1}^{*}M_{2}+\beta _{2}^{*}M_{4}+d^{*})M_{1} -(\sigma ^{*}+d^{*}+d^{*}_{1})M_{2} ]\frac{t^{p}}{\Gamma (p+1)}}, \\ {\mathbf{R}_{1}(t)} = { [\sigma ^{*}M_{2}-d^{*}M_{3} ]\frac{t^{p}}{\Gamma (p+1)}}, \\ {\mathbf{W}_{1}(t)} = { [\alpha ^{*}M_{2}-\eta ^{*}M_{4} ]\frac{t^{p}}{\Gamma (p+1)}}, \end{cases}\displaystyle \\& q^{2}: \textstyle\begin{cases} {\mathbf{S}_{2}(t)} = [\Pi ^{*}\frac{t^{p}}{\Gamma (p+1)} ]- [\beta ^{*}_{1}(k_{11}M_{1}+g_{11}M_{2}) \\ \hphantom{{\mathbf{S}_{2}(t)} =} {} +\beta ^{*}_{2}(v_{11}M_{1}+g_{11}M_{4})+d^{*}g_{11} ]\frac{t^{2p}}{\Gamma (2p+1)}, \\ {\mathbf{I}_{2}(t)} = [\beta ^{*}_{1}(k_{11}M_{1}+g_{11}M_{2})+\beta ^{*}_{2}(v_{11}M_{1} +g_{11}M_{4}) \\ \hphantom{{\mathbf{I}_{2}(t)} =} {}+d^{*}g_{11}-(\sigma ^{*}+d^{*}+d^{*}_{1})M_{2} ]\frac{t^{2p}}{\Gamma (2p+1)}, \\ {\mathbf{R}_{2}(t)} = { [\sigma ^{*}k_{11}-d^{*}u_{11} ]\frac{t^{2p}}{\Gamma (2p+1)}}, \\ {\mathbf{W}_{2}(t)} = { [\alpha ^{*}k_{11}-\eta ^{*}v_{11} ]\frac{t^{2p}}{\Gamma (2p+1)}}, \end{cases}\displaystyle \end{aligned}$$

and

$$\begin{aligned}& q^{3}:\textstyle\begin{cases} {\mathbf{S}_{3}(t)}= { [\Pi ^{*}\frac{t^{p}}{\Gamma (p+1)} ]}-{ [(M_{1}g_{11}k_{11}+M_{1} \Pi ^{*})\beta _{1}+(v_{11}g_{11}+M_{4}+d^{*}\Pi ^{*})\beta _{2} ]}\frac{t^{2}p}{\Gamma (2p+1)} \\ \hphantom{{\mathbf{S}_{3}(t)}=}{} -{ [\beta _{1}M_{1}k_{22}-\beta _{2}g_{22}+\beta _{2}M_{1}v_{22}- \beta _{2}M_{4}g_{22}-d^{*}g_{2} ]}\frac{t^{3}p}{\Gamma (3p+1)}, \\ {\mathbf{I}_{3}(t)}= { [\beta _{1}M_{1} g_{11} k_{11}-\beta _{1}M_{1}\Pi ^{*}-\beta _{2}v_{11}g_{11}-\beta _{2}M_{4} -d^{*} \Pi ^{*} ]} \frac{t^{2p}}{\Gamma (2p+1)} \\ \hphantom{{\mathbf{I}_{3}(t)}=}{} + [\beta _{1}M_{1}k_{22}+\beta _{2}g_{22}-\beta _{2}M_{1}v_{22}- \beta _{2}M_{4}g_{22}+d^{*}g_{2}- (\sigma ^{*}+d^{*} \\ \hphantom{{\mathbf{I}_{3}(t)}=}{}+d^{*}_{1} ) ]{\frac{t^{3p}}{\Gamma (3p+1)}}, \\ {\mathbf{R}_{3}(t)}= \mathrm{ (\sigma ^{*}k_{11}-d^{*}u_{11} )}{ \frac{t^{3p}}{\Gamma (3p+1)}}, \\ \mathbf{W}_{3}(t)= \mathrm{ (\alpha k_{11}-\eta v_{11} )}{ \frac{t^{3p}}{\Gamma (3p+1)}}, \end{cases}\displaystyle \end{aligned}$$

where the unknown terms are

$$\begin{aligned}& g_{11} = \Pi ^{*}-\beta _{1}^{*}M_{1}M_{2}- \beta _{2}^{*}M_{1}M_{4}-d^{*}M_{1}, \\& k_{11} = \beta _{1}^{*}M_{1}M_{2}+ \beta _{2}^{*}M_{1}M_{4}+d^{*}M_{1} -\bigl( \sigma ^{*}+d^{*}+d^{*}_{1} \bigr)M_{2}, \\& u_{11} = \sigma ^{*}M_{2}-d^{*}M_{3}, v_{11}=\alpha ^{*}M_{2}- \eta ^{*}M_{4}, \\& g_{22} = - \bigl[\beta ^{*}_{1}(k_{11}M_{1}+g_{11}M_{2}) +\beta ^{*}_{2}(v_{11}M_{1}+g_{11}M_{4})+d^{*}g_{11} \bigr], \\& k_{22} = \beta ^{*}_{1}(k_{11}M_{1}+g_{11}M_{2})+ \beta ^{*}_{2}(v_{11}M_{1} +g_{11}M_{4})+d^{*}g_{11}-\bigl(\sigma ^{*}+d^{*}+d^{*}_{1}\bigr)M_{2}. \end{aligned}$$

It should be noted that, in the methods discussed above, the solutions of the considered model are obtained in the form of infinite-series solutions that converge rapidly to an accurate solution of the system (for details, see [42–46] and the references therein).

## Numerical results and discussion

**Figure 1:**
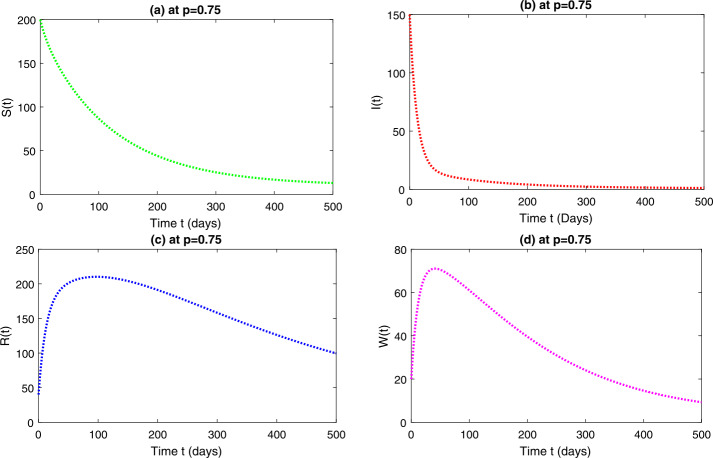
Dynamical behavior of $\mathbf{S}_{h}(t)$, $\mathbf{I}_{h}(t)$, $\mathbf{R}_{h}(t)$ and $\mathbf{W}(t)$ at fractional-order $p=0.75$

**Figure 2:**
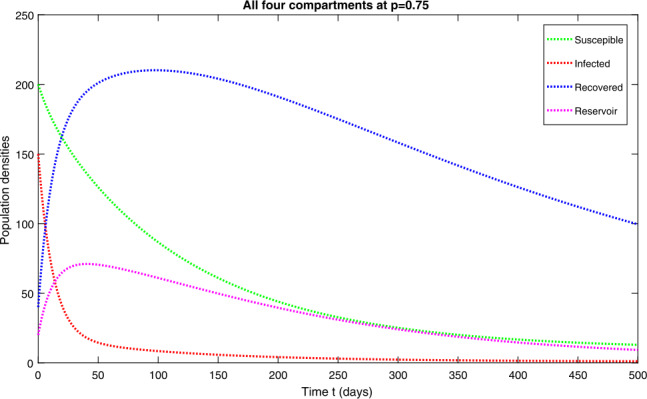
Dynamical behavior of $\mathbf{S}_{h}(t)$, $\mathbf{I}_{h}(t)$, $\mathbf{R}_{h}(t)$ and $\mathbf{W}(t)$ at fractional-order $p=0.75$

**Figure 3:**
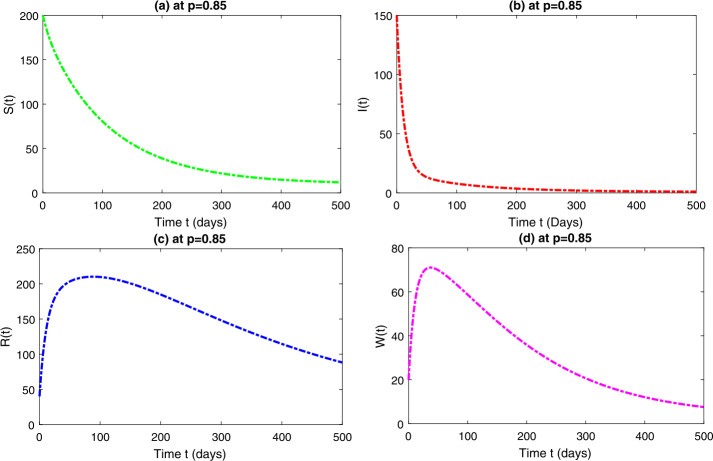
Dynamical behavior of $\mathbf{S}_{h}(t)$, $\mathbf{I}_{h}(t)$, $\mathbf{R}_{h}(t)$ and $\mathbf{W}(t)$ at fractional-order $p=0.85$

**Figure 4:**
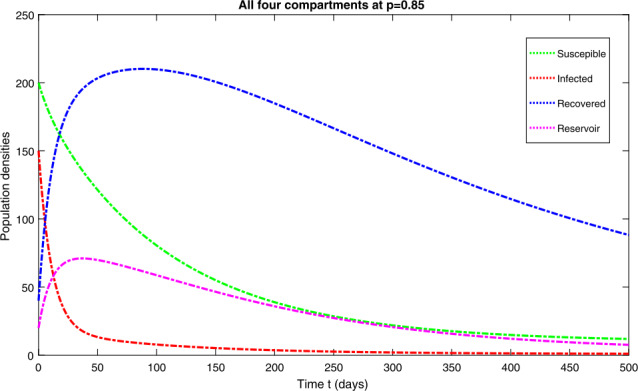
Dynamical behavior of $\mathbf{S}_{h}(t)$, $\mathbf{I}_{h}(t)$, $\mathbf{R}_{h}(t)$ and $\mathbf{W}(t)$ at fractional-order $p=0.85$

**Figure 5:**
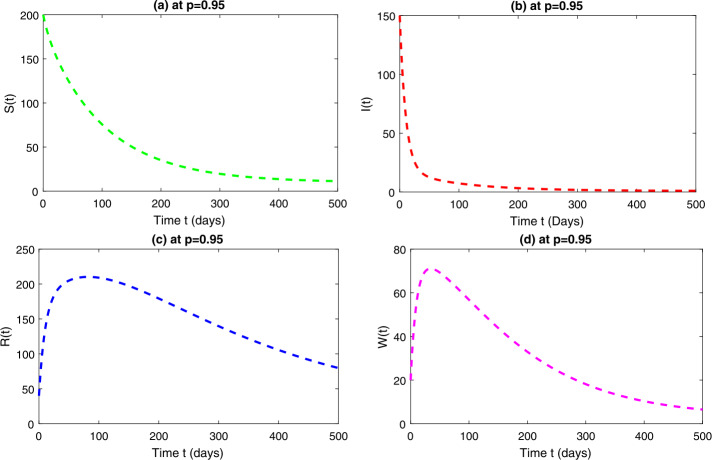
Dynamical behavior of $\mathbf{S}_{h}(t)$, $\mathbf{I}_{h}(t)$, $\mathbf{R}_{h}(t)$ and $\mathbf{W}(t)$ at fractional-order $p=0.95$

**Figure 6:**
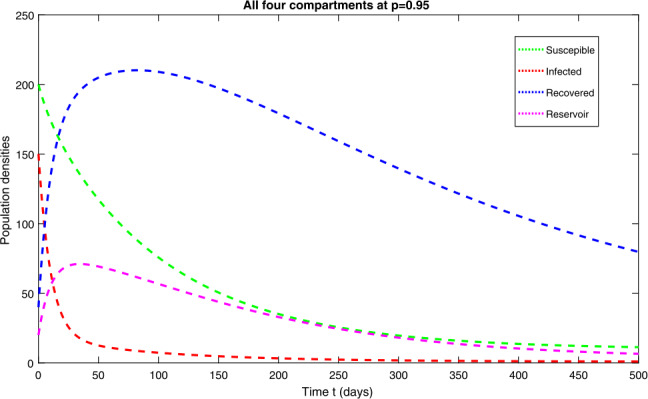
Dynamical behavior of $\mathbf{S}_{h}(t)$, $\mathbf{I}_{h}(t)$, $\mathbf{R}_{h}(t)$ and $\mathbf{W}(t)$ at fractional-order $p=0.95$

**Figure 7:**
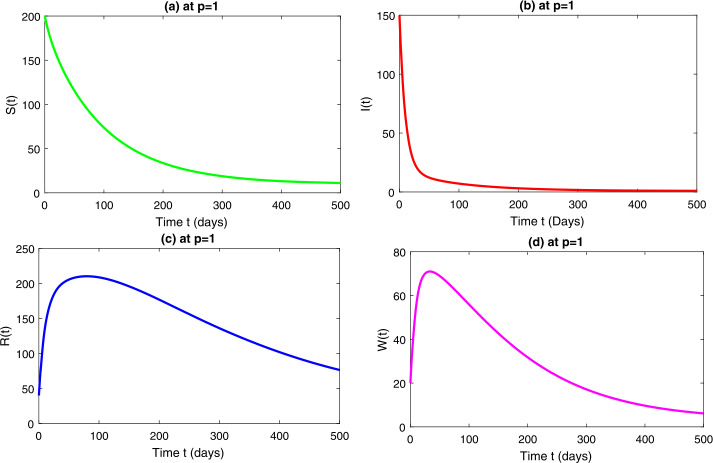
Dynamical behavior of $\mathbf{S}_{h}(t)$, $\mathbf{I}_{h}(t)$, $\mathbf{R}_{h}(t)$ and $\mathbf{W}(t)$ at integer order $p=1$

**Figure 8:**
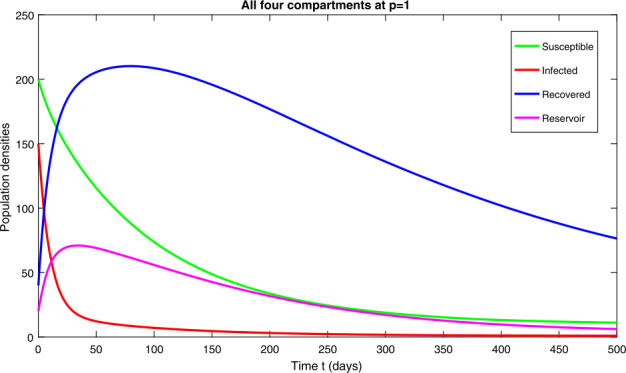
Dynamical behavior of $\mathbf{S}_{h}(t)$, $\mathbf{I}_{h}(t)$, $\mathbf{R}_{h}(t)$ and $\mathbf{W}(t)$ at integer order $p=1$

In this section, we study the numerical solutions of the considered model (3) obtained from the proposed techniques. The computational findings are based on both qualitative and quantitative analysis because some of the parameters are estimated from the available sources, while others are assumed with biological feasibility. By using LADM on the first few terms of every compartment of model (3) for parameters $\Pi ^{*}= 0.093907997$, $\beta _{1}^{*}= 0.00005$, $\beta _{2}^{*}=0.0000000123$, $d^{*}=0.009567816$, $\sigma ^{*}=0.09871$, $d_{1}^{*}= 0.00404720925$, $\alpha ^{*}= 0.0398$, $\eta ^{*}= 0.01$. For the given four classes, we present the numerical results for different fractional-orders in Figs. 1–8.

*Case-I*: In Figs. 1(a)–(d), the four quantities have been simulated for time 0–500. Figure 1(a) is the susceptible class representing exponential decay and converges to the stable state with the passage of time for various arbitrary orders at *p*. Figure 1(b) is the infected class that shows a rapid decrease at various fractional-orders. Figure 1(c) is the recovered class at different fractional-order at *p*. This group represents an exponential growth at the beginning as large numbers of individuals have been infected and by hospitalization and using precautionary measures, they recovered quickly with the passage of time. One can see that, with the passage of time, the recovery rate reaches an ultimate point and then becomes stable. Figure 1(d) is the reservoir group representing a small increase at the initial stage and then attaining the highest point. It is observed that the reservoir class decreases for a short interval of time and then becomes stable. The mutual representation of all four quantities at $p=0.75$ is given in Fig. 2.

*Case-II*: Fig. 3 represents the four quantities of model (3) at fractional-order $p=0.85$, which shows a similar behavior to that obtained at $p=0.75$. It should be noted that, as we increase the fractional-order, the behavior of the curves are getting closer to the integer order with $p=1$. Figure 4 represents the combined effect of the four classes of model (3) at $p=0.85$.

*Case-III:* Fig. 5 is the representation of all four quantities of model (3) at fractional-order $p=0.95$, which shows similar behavior to that obtained in *case-II*. One can observe that, as the order increases, the behavior of the curves is getting closer to the integer-order value at $p=1$. Figure 6 is the combined behavior of all the four quantities of (3) at $p=0.95$.

*Case-IV:* In Fig. 7, representing model (3) at integer order $p=1$, we see a similar behavior as discussed in *case-III*. Figure 8 is the combined behavior of all the four classes of (3) at $p=1$.

The obtained results reveal that the various compartmental populations of the proposed model have increased in the first few days, and then start decreasing with the passage of time, see Figs. 1–7. It should be also noted that the infected individuals disappear after 300 days, as presented in Fig. 1(b), while the reservoir will exist for many years, as seen in Fig. 1(d). This clearly suggests that strict SOPSs and proper control measures need to be followed to avoid spreading of the disease.

## Summary

In this work, we have investigated a fractional model for COVID-19 in the Caputo sense. The considered model is studied for the analytical solutions using LADM and HPM and we calculated the series solutions for the first four terms. It is observed that both methods present similar results for the considered system. It is also observed that approximations provide better results than classic derivatives. The numerical simulations are provided with two different sets of initial conditions that shows convergence to the same equilibrium points for all the four compartments of the considered model. Finally, one can see good agreements between the numerical and approximations with realistic interpretations for the system at random fractional-order.

## Data Availability

The data that support the findings of this study are included in the manuscript.
